# 

**Published:** 1896-07

**Authors:** 


### NOTICES.

### American Dental Association.

Dr. H. J. Burkhart requests us to announce that at the meeting of the American Dental Association at Saratoga, rooms will be furnished free of charge by the proprietors of the Grand Union Hotel, which has been chosen headquarters for Faculties and Examiners' Associations, School of Technics, etc., also rooms for the Section meetings.

### National Association of Dental Faculties.

The annual meeting of the National Association of Dental Faculties will be held at Saratoga Springs on Saturday, August 1, 1896, at 10 o'clock, A. M. The Executive Committee will meet on Friday, July 31st, at 10 o'clock, A. M. Those having matters to bring before this committee will do well to bear this meeting in mind. The work of this committee is preparatory to that of the general meeting. All communications requiring the attention of the Executive Committee should be sent to the chairman.

DR. J. TAFT, *Chairman of Executive Committee*,
Berkshire Building, Cincinnati, Ohio.

DR. LOUIS OTTOFY, *Secretary*,
Masonic Temple, Chicago, Illinois.

### American Dental Association.

The annual meeting of the American Dental Association will be held at Saratoga Springs, N. Y., July 31st to August 7, 1896. The regular meetings of the American will begin Tuesday, August 4th.

The Grand Union Hotel has been selected as the headquarters, and the meetings will be held there. Ample space for Committee-rooms and Exhibits has been secured. The ball-room and Club-house, connecting with ball-room, have also been placed at our disposal.

The railroad arrangements have not yet been completed, but we expect to secure the usual fare-and-a-third rate. Tickets good three days before date of meeting, and three days after the close. Dentists should pay full-fare going and take a receipt therefor, as it will be impossible to secure the one-third rate in returning unless you hold such receipt.

J. N. Crouse, *Chairman Executive Committee.*

### American Dental Association.

The American Dental Association will hold its thirty-sixth annual session at Saratoga Springs, N. Y., commencing at 10 o'clock, A. M., on Tuesday, August 4, 1896.

Geo. H. Cushing, *Recording Secretary.*

### The National Association of Dental Examiners.

The twelfth annual session will be held at Saratoga Springs, N. Y., commencing at 10 o'clock A. M. Monday, August 3d, and continuing in session during the proceedings of the American Dental Association.

It is earnestly requested that all State and territorial boards will send delegates.

Chas. A. Meeker,
Secretary and Treasurer,
29 Fulton street, Newark, N. J.

### Twelfth International Medical Congress.

The Twelfth International Congress of Medicine will be held at Moscow, Russia, August 19–26, 1897.

President, J. F. Klein; Secretary, F. F. Erismann. The fee is $5.00, which should be sent to Prof. N. Zilatow, treasurer of the Congress. The Congress is composed of medical men only, but persons having a scientific title, are admitted to the sessions of the Congress as extraordinary members. Veterinary Surgeons, Pharmarists and Dentists are classed as extraordinary members. The work of the Congress comprises twelve sections. The ninth section contains surgery, which includes diseases of the larynx, the ear and the teeth.

### Book Notice.

It will be a matter of interest to the dental profession to learn that R. L. Polk & Co., of Detroit, Mich., have in preparation and will soon issue the second edition of the DENTAL REGISTER for the United States. The last edition—the most complete work of its kind for dentists ever published—came out in 1893. So many members have come into the profession since that time, so many have dropped out, and so many have changed location as to render a new and corrected edition a necessity for all who have occasion to refer to such a work. The former issue is a very full and accurate one up to the time of its publication, and is an earnest of the completeness of the forthcoming volume. Such a work as this is well-nigh a necessity for every dentist who is a member of a dental society, or who has any considerable correspondence with his professional brethren. The work is promised at an early date. It will contain the names of about twenty thousand dentists with graduation particulars, list of colleges, laws, and other valuable information relating to the profession. We can most heartily recommend this work to every progressive dentist.

### THE DENTAL REGISTER,

**A MONTHLY MAGAZINE DEVOTED TO THE INTERESTS OF THE**

### DENTAL PROFESSION.

*Terms Reduced to $2.00 Per Year. Single Copies, 25 Cents.*

**EDITED BY PROF. J. TAFT, D.D.S.**

—AND—

**Published by SAM'L A. CROCKER & CO., Ohio Dental and Surgical Depot.**

The volume commences in January and closes in December.

The REGISTER being issued monthly is always fresh, therefore Indispensable to the practitioner who desires to keep posted up with the new inventions and improvements which are daily developed. Neither expense nor effort will be spared to give value and variety to its contents. Articles will be illustrated with well executed engravings whenever they will add to the interest or assist to explanation.

Its extensive circulation and frequency of issue make it one of the BEST DENTAL ADVERTISING MEDIUMS in the United States.

All subscriptions must begin with January or July.

Local or special notices, five lines or less, will be inserted for $3 each insertion; over five lines, 50 cts. per line.

All advertising bills payable in advance, or at furthest, after the issue of the first number containing the advertisement. All transient advertisements to be paid for in advance.

☞CONTRIBUTIONS SOLICITED.

Exclusively Editorial Communications should be addressed to the Editor.

The latest number of the REGISTER may be ordered with or without other goods [illegible] time, and all letters should be addressed to [illegible]

